# Organization of Sensory Feature Selectivity in the Whisker System

**DOI:** 10.1016/j.neuroscience.2017.09.014

**Published:** 2018-01-01

**Authors:** Michael R. Bale, Miguel Maravall

**Affiliations:** Sussex Neuroscience, School of Life Sciences, University of Sussex, Brighton BN1 9QG, United Kingdom

**Keywords:** BC, barrel cortex, PWA, primary whisker afferents, S1, primary somatosensory cortex, VPM, ventral posterior medial thalamic nucleus, S2, secondary somatosensory cortex, PPC, posterior parietal cortex, M1, primary motor cortex, vibrissa, coding, receptive field, information, somatotopy, map

## Abstract

Our sensory receptors are faced with an onslaught of different environmental inputs. Each sensory event or encounter with an object involves a distinct combination of physical energy sources impinging upon receptors. In the rodent whisker system, each primary afferent neuron located in the trigeminal ganglion innervates and responds to a single whisker and encodes a distinct set of physical stimulus properties – features – corresponding to changes in whisker angle and shape and the consequent forces acting on the whisker follicle. Here we review the nature of the features encoded by successive stages of processing along the whisker pathway. At each stage different neurons respond to distinct features, such that the population as a whole represents diverse properties. Different neuronal types also have distinct feature selectivity. Thus, neurons at the same stage of processing and responding to the same whisker nevertheless play different roles in representing objects contacted by the whisker. This diversity, combined with the precise timing and high reliability of responses, enables populations at each stage to represent a wide range of stimuli. Cortical neurons respond to more complex stimulus properties – such as correlated motion across whiskers – than those at early subcortical stages. Temporal integration along the pathway is comparatively weak: neurons up to barrel cortex (BC) are sensitive mainly to fast (tens of milliseconds) fluctuations in whisker motion. The topographic organization of whisker sensitivity is paralleled by systematic organization of neuronal selectivity to certain other physical features, but selectivity to touch and to dynamic stimulus properties is distributed in “salt-and-pepper” fashion.

## Highlights

• Neurons in the whisker system are selective to spatial and dynamical properties – features – of sensory stimuli.
• At each stage of the pathway, different neurons encode distinct features, generating a rich population representation.
• Whisker touch is robustly represented; neurons respond to touch-driven fast fluctuations in forces at the whisker base.
• Cortical neurons have more complex and context-dependent selectivity than subcortical, e.g., to collective whisker motion.
• Understanding how these signals are integrated to construct whisker-mediated percepts requires further research.

## Introduction

Work initially conducted in the visual system and carried out over several decades has shown that neurons in sensory pathways respond to specific physical properties of a stimulus – for instance, light of a certain wavelength or sound of a certain frequency, coming from a particular region of space over a given time window. We refer to these properties that neurons are selective to, and convey information about, as stimulus “features”. In the whisker system, neurons are selective to the parameters that describe interactions between whiskers and the objects that interfere with their motion. For a surprisingly long time, these interactions were poorly understood, so that the structure of the inputs to the whisker pathway was effectively unknown. However, recent advances in methods for tracking whiskers, identifying cell types, and modeling the array of forces that come into play during whisker deflections, have brought within sight a comprehensive understanding of the physical input to primary whisker afferents (PWAs) and of the responses of at least some categories of PWA. This progress is reviewed in detail by Campagner et al. in this Special Issue (Campagner et al., 2018).

In brief, PWAs are selective for both whisker identity – because each innervates a single whisker – and for mechanical forces acting on the whisker follicle as a result of whisker motion and bending. Mechanotransduction reflects the particular array of forces and moments at play at the whisker base. During different modes of whisker exploration, these parameters are in close correspondence with kinematic properties of whisker motion, with the existence of whisker contacts and with the amplitude and phase of whisking itself (Szwed et al., 2003, Szwed et al., 2006, Pammer et al., 2013, Campagner et al., 2016, Bush et al., 2016). Different PWAs extract distinct combinations of forces and moments (Bale et al., 2013, Chagas et al., 2013, Campagner et al., 2016, Bush et al., 2016). Because of this diversity in the features represented by PWAs as a population, the trigeminal ganglion conveys a high-resolution, detailed picture of fluctuations in whisker motion. The features are typically brief (corresponding to sensing fluctuations over ∼1 ms) (Stuttgen et al., 2008, Lottem and Azouz, 2008, Lottem and Azouz, 2009, Maravall et al., 2013, Bale et al., 2013, Chagas et al., 2013, Campagner et al., 2016, Bush et al., 2016, Severson et al., 2017), implying that the representation of whisker motion also has very high temporal resolution.

Here we review work on how this sensory information is used and transformed along the whisker pathway. Beginning with classic work, we describe analyses of feature selectivity performed under controlled stimulation and compare those with more recent studies that examine responses in awake animals actively sensing their environment. We address the following questions. What sensory information do neurons in the pathway communicate about a tactile stimulus? Are there systematic changes in feature selectivity from stage to stage? How are neurons that encode different stimulus features distributed at each stage in the pathway? And how does feature selectivity vary with cell type?

## Analysis of sensory feature selectivity using controlled stimulation

### Determining elementary stimulus parameters to which neurons are sensitive

In pioneering studies of feature selectivity in the whisker system, researchers delivered whisker deflection stimuli passively to anaesthetized rodents and determined neuronal preferences by counting the number of evoked spikes (Zucker and Welker, 1969, Waite, 1973, Shipley, 1974, Axelrad et al., 1976, Simons, 1978). Neurons were found to respond selectively to physical features that can be classified into three different types. First and most obviously, neurons were selective for whisker identity, a feature made more striking by the somatotopic representation of whisker identity in the primary somatosensory cortex (S1), the ventral posterior medial nucleus of the thalamus (VPM) and the principal nucleus of the brainstem (Woolsey and Van Der Loos, 1970, Shipley, 1974, Van Der Loos, 1976, Ma, 1991). Second, neurons were tuned to the angular direction of a deflection in the plane orthogonal to the whisker follicle, relative not just to the principal (rostrocaudal) axis of whisking but also to the dorsoventral axis – so-called directional selectivity (Zucker and Welker, 1969, Waite, 1973, Shipley, 1974, Axelrad et al., 1976, Simons, 1978, Gibson and Welker, 1983a, Gibson and Welker, 1983b, Lichtenstein et al., 1990). Finally, beyond spatial features, neurons were also selective for different kinematic features of whisker dynamics – that is, the amplitude of whisker deflection and its temporal derivatives. As might be expected, the larger the amplitude the larger the response (Zucker and Welker, 1969, Gibson and Welker, 1983a, Gibson and Welker, 1983b, Simons and Carvell, 1989). Neurons were also sensitive to the speed of deflection (Gibson and Welker, 1983a, Gibson and Welker, 1983b, Shoykhet et al., 2000), a parameter potentially transmitted selectively to the barrel cortex (BC), as responses to slower speeds are dampened by feedforward inhibition (Pinto et al., 2000, Pinto et al., 2003, Wilent and Contreras, 2004, Yu et al., 2016).

A later generation of studies attempted to identify more systematically the nature and temporal duration of the dynamical features of passively driven whisker motion to which neurons are selective. Reverse correlation techniques relating the occurrence of a spiking response to earlier structure in the stimulus were originally conducted in the visual and auditory pathways (de Boer and Kuyper, 1968), as reviewed in (Sharpee, 2013, Aljadeff et al., 2016). Their initial application to the whisker system – specifically, VPM and BC – revealed sensitivity to fast fluctuations in whisker motion over time: neurons were sensitive to kinematic parameters such as speed, amplitude, acceleration or combinations thereof (Maravall et al., 2007, Petersen et al., 2008, Estebanez et al., 2012). Importantly, different neurons within each processing stage had preferences for differing whisker kinematic features (Petersen et al., 2008).

### Comparison of processing stages in the whisker pathway

Later analyses have extracted feature selectivity at several stages in the whisker pathway in a manner that allows for comparison between them. Variants of reverse correlation analysis have predicted spiking responses as a function of stimulus parameters in the trigeminal ganglion (Jones et al., 2004, Arabzadeh et al., 2005, Bale et al., 2013, Maravall et al., 2013, Theis et al., 2013, Chagas et al., 2013), VPM (Petersen et al., 2008, Maravall et al., 2013) and BC (Maravall et al., 2007, Estebanez et al., 2012). These studies have found that feature selectivity across stages can be understood within a common framework where neurons respond to a particular kinematics pattern over time.

However, work has also identified systematic differences across stages. Subcortical neurons are typically selective for a single, short-duration feature (e.g., velocity on a timescale of <10 ms) (Petersen et al., 2008, Bale et al., 2013). In contrast, and analogous to the situation in other sensory pathways, cortical neurons typically have more complex feature selectivity. First, BC neurons typically display sensitivity to multiple features rather than a single one – i.e. responses are predicted by the conjoint “presence” in the stimulus of multiple whisker fluctuation waveforms (Maravall et al., 2007, Estebanez et al., 2012). Second, sensitivity of BC neurons to individual features often displays substantial nonlinearities: for example, neurons are often sensitive to the absolute value of a parameter, such as speed – which is invariant to direction – as opposed to signed velocity (Arabzadeh et al., 2005, Maravall et al., 2007, Estebanez et al., 2012). Stimulus features are diverse, i.e., heterogeneous across neurons, at every stage where they have been assessed, implying a rich representation of whisker dynamics at each stage. Thus, populations of neurons throughout the whisker pathway, particularly in BC, do not encode a single stimulus physical parameter or dimension; rather, they represent a mosaic of features defined across multiple dimensions.

### Sensitivity to spatially patterned stimulation

The above findings suggest that BC neuronal populations could have the potential to provide a detailed code for representing complex whisker stimuli. This possibility has recently begun to be addressed with the use of stimulators capable of deflecting multiple whiskers separately, thus delivering complex spatiotemporal patterns of stimulation. This approach has revealed cortical neurons specifically sensitive to correlated or uncorrelated motion of multiple whiskers (Estebanez et al., 2012, Estebanez et al., 2016). These results build upon earlier reports of nonlinear integration of the motion of multiple whiskers (reviewed in (Petersen et al., 2009)) and indicate that cortical neurons may construct “apparent motion” from sequential deflection of adjacent whiskers, potentially underpinning the ability to extract information about object curvature or shape from exploration mediated by multiple whiskers (Jacob et al., 2008, Hobbs et al., 2015) (see *Organization of selectivity to spatial features* for further discussion). This sensitivity to multiple-whisker motion appears specifically enhanced in cortex as compared to VPM (Hirata and Castro-Alamancos, 2008, Ego-Stengel et al., 2012). Thus, cortical neurons integrate over space (i.e. multiple whiskers) more strongly than subcortical cells. This active area of research is reviewed by Estebanez et al. in this Special Issue (Estebanez et al., 2018).

### Sensitivity to temporally patterned stimulation: encoded features and temporal integration

In a scanning tactile modality such as the whisker system, integration over time is also functionally relevant: exploring an object by whisker scanning or simply running along walls or tunnels will generate a series of fluctuations in whisker dynamics, concatenated over time (Wolfe et al., 2008, Jadhav et al., 2009, Jenks et al., 2010, Sofroniew et al., 2014, Sofroniew and Svoboda, 2015). Identifying a texture may involve integrating over such fluctuations, i.e. being selective to the aggregate pattern of fluctuations accumulated over a certain time window. This capacity is present in rodents, which can be trained to discriminate a temporal pattern of whisker fluctuations occurring in a particular order (Bale et al., 2017).

Windows for temporal integration can be assessed by measuring the time courses of preferred features. These correspond to the duration of the fluctuations in whisker motion encoded by a neuron’s response. In BC neurons, these windows are in the range of several tens of (∼40–50) milliseconds (Maravall et al., 2007, Estebanez et al., 2012), which compare with a few milliseconds in VPM (Petersen et al., 2008). This increase in duration from VPM to BC denotes a loss in bandwidth or temporal resolution; however, it is too short for integrating over multiple whisks (each of which typically lasts around 50 ms) or multiple “stick–slip” sensory events characteristic of texture exploration (Von Heimendahl et al., 2007, Ritt et al., 2008, Wolfe et al., 2008, Jadhav et al., 2009). These findings are consistent with results from complementary methods for assessing integration times (Stuttgen and Schwarz, 2010, Waiblinger et al., 2015a, Mcguire et al., 2016, Pitas et al., 2016). Collectively, they suggest that the timescale of integration for most BC neurons is under 100 ms, lower than that needed to account for behavior (Mcguire et al., 2016, Fassihi et al., 2017), implying that higher cortical areas must carry out further temporal integration. Several cortical areas with direct projections from BC (Aronoff et al., 2010, Zakiewicz et al., 2014) are natural candidates for this temporal integration of sensory information (Yamashita et al., 2013), including secondary somatosensory cortex (S2) (Yang et al., 2016, Kwon et al., 2016), posterior parietal cortex (PPC) (Mohan et al., 2017) and primary motor cortex (M1) (Fassihi et al., 2017). A role for BC as an encoder primarily of current rather than time-integrated or averaged stimulus parameters (Pitas et al., 2016) is consistent with observations in S1 of other species (Romo and de Lafuente, 2013), suggesting a common principle across tactile pathways.

### Neuronal tuning: adaptation and gain control

How do neurons modulate their response to the presence of their preferred feature – how sharply are they tuned? Reverse correlation studies have shown subcortical neurons are tuned so as to provide a direct, faithful representation of feature magnitude – i.e. responses increase monotonically and roughly proportionally to the magnitude of the stimulus as convolved with the preferred feature (Petersen et al., 2008, Maravall et al., 2013, Bale et al., 2013). In further contrast to early subcortical stages, where neurons are often sensitive to stimulus magnitude in absolute terms (Maravall et al., 2013), neurons in BC are tuned in a manner that suggests responsiveness to the presence of tactile events that exceed background by a significant proportion: that is, they respond when the presence of the preferred feature in the stimulus diverges from the background by a comparatively large amount. This sensitivity relative to ongoing statistics, or adaptive gain rescaling, enables detection of events against silence and of salient fluctuations in ongoing stimulation (Maravall et al., 2007). This quality is a form of adaptive coding (Ollerenshaw et al., 2012, Ollerenshaw et al., 2014, Davies et al., 2012) and parallels neurons in other cortical sensory areas (Ringach and Malone, 2007, Carandini and Heeger, 2012).

The results of this section are summarized in Table 1.

**Table 1:** Comparative overview of feature selectivity properties of principal neurons at certain stages of the whisker pathway

	Sensitivity to multiple whiskers	Sensitivity to multiple dynamical features	Encoding of whisker motion direction	Encoding of correlation in whisker motion	Temporal precision (jitter order of magnitude)	Temporal integration window	Heterogeneity in feature selectivity across neurons	Adaptive context sensitivity
PWA	✗	Minority	Strongest	✗	10^−2^ ms	1–10 ms	✓	Some neurons display fixed gain, others rescale their gain
VPM	✓	Minority	Strong	?	10^−1^ ms	2–20 ms	✓	Diverse: some neurons are fixed gain, others rescale gain, others vary shape of tuning curve
S1	✓	Majority	Weaker	✓	10^0^ ms	10–50 ms	✓	Majority with gain rescaling

PWA, primary whisker afferents; VPM, neurons in the thalamic ventral posterior medial nucleus; S1, neurons in the primary somatosensory barrel cortex.

## From controlled stimulation to awake sensing: feature selectivity across modes of stimulation and exploration

### Feature selectivity can depend on context

The above results (Table 1) relied on passive whisker deflection using arbitrary stimulus waveforms to characterize feature selectivity across the pathway. Studies have tested the general applicability of these findings by assessing whether feature selectivity changes under more naturalistic forms of stimulation, starting with artificial (passive) playback of natural whisker motion trajectories (Arabzadeh et al., 2005, Lottem and Azouz, 2011, Bale et al., 2013, Bale et al., 2015). To reproduce the mechanical configuration experienced by whisker follicles, and consequently by mechanoreceptors, during natural whisker exploration, an array of studies of neuronal coding has used “electrical whisking” – stimulation of the buccal branch of the facial nerve to achieve whisker motion – (Zucker and Welker, 1969, Brown and Waite, 1974, Szwed et al., 2003, Arabzadeh et al., 2005, Hipp et al., 2006, Lottem and Azouz, 2011, Wallach et al., 2016), or monitoring of active motion in awake animals performing discrimination tasks (O'connor et al., 2010a, O'connor et al., 2010b, O'connor et al., 2013, Petreanu et al., 2012, Chen et al., 2013, Hires et al., 2015, Peron et al., 2015, Sofroniew et al., 2015, Bush et al., 2016, Campagner et al., 2016, Severson et al., 2017). Overall, these studies have revealed that some feature selectivity properties are highly robust across conditions, but have also shown that monitoring of active motion is necessary to disambiguate the true nature of the features to which whisker pathway neurons are selective.

### Robust aspects of feature selectivity: sensitivity to touch and diversity of tuning properties

One robust finding, verified across experimental paradigms in behaving animals, is that whisker touch (contact) – with associated changes in whisker curvature and thus in the pattern of forces at the follicle – is the principal sensory driver of responses in many BC neurons (Crochet and Petersen, 2006, Von Heimendahl et al., 2007, Curtis and Kleinfeld, 2009, Jadhav et al., 2009, O'connor et al., 2010b, Chen et al., 2013, Sachidhanandam et al., 2013, Hires et al., 2015, Peron et al., 2015). Further, touch also drives responses in subsets of neurons within M1 (Huber et al., 2012, Petreanu et al., 2012, Chen et al., 2017). In layer 4 of BC, whisker touches evoke precisely timed spikes (Hires et al., 2015), enforced by strong feedforward inhibition that gates thalamic input according to its degree of synchrony (Pinto et al., 2000, Pinto et al., 2003, Bruno and Simons, 2002, Gabernet et al., 2005, Daw et al., 2007, Cruikshank et al., 2007, Yu et al., 2016). One study compared responses to whisker touch in the same neurons under active exploration during wakefulness and passive stimulation during anesthesia (Peron et al., 2015). Surprisingly, in this study most neurons responding to touch did so only in either of the two conditions, not both.

Although characterization of feature selectivity during awake exploration in different tasks remains incomplete, touch is a key driver of responses across tasks. In a situation where whiskers move against a smooth object in order to localize it (O'connor et al., 2010a, O'connor et al., 2010b), the presence and number of touches – as encoded by variations in response spike rate – is the principal behavioral cue (O'connor et al., 2013), and response variability induced by stick–slip events is minor. On the other hand, the strength of the touch response is likely to be modulated by specific feature selectivity under certain behaviorally relevant conditions, e.g., when the animal is faced by a wall or textured object. Exploration of rough objects and textures evokes sequences of dynamic stick–slip events whose nature is encoded through sensitivity to the features described earlier (Arabzadeh et al., 2005, Jadhav et al., 2009, Zuo et al., 2011, Chen et al., 2015, Waiblinger et al., 2015a, Waiblinger et al., 2015b, Bale et al., 2015).

The finding that neurons located at the same stage of processing display diverse sensory feature preferences is robust across experimental conditions. It has been replicated in all paradigms, ranging from recordings of PWAs during passive replay of naturalistic stimulus waveforms (Bale et al., 2013) to BC recordings in animals performing sensory discrimination tasks (Von Heimendahl et al., 2007, Jadhav et al., 2009, O'connor et al., 2010b, Petreanu et al., 2012, Safaai et al., 2013, Chen et al., 2013, Clancy et al., 2015, Peron et al., 2015, Sofroniew et al., 2015), and even in axons projecting to BC from M1 (Petreanu et al., 2012).

An important difference between assessment of dynamical feature selectivity during passive and active sensing is that the former cannot properly distinguish between selectivity to kinematic properties (whisker position or angle and their temporal derivatives) or mechanical properties (forces acting at the base of the whisker shaft). This is because under certain conditions including passive stimulation, both classes of properties are strongly correlated or coupled (Campagner et al., 2016, Campagner et al., 2018, Bush et al., 2016). To disambiguate these features, it is necessary to monitor whisker motion during active sensing. Under these conditions, PWA responses primarily encode mechanical features, consistent with the nature of mechanotransduction as reflecting the array of forces and moments acting in the vicinity of the sensor. It is thus reasonable to assume that selectivity to mechanical properties is handed down from PWAs to the rest of the pathway.

## Spatial organization of feature selectivity

The discovery of sensory and motor homunculi decades ago and consequent identification of topographic maps in the human somatosensory system, gave rise to the compelling idea that the brain organizes information according to maps – whereby the functional role of a neuron, and its tuning to stimulus properties, can be predicted by its location. Indeed, whisker somatotopy is perhaps the most striking large-scale property of the lemniscal whisker pathway’s organization (Woolsey and Van Der Loos, 1970, Welker and Woolsey, 1974, Welker, 1976). At every stage beyond the trigeminal ganglion and up to S1, neurons responding to the same whisker follicle are located near to each other, and neurons responding to adjacent whiskers are in adjacent areas. Given this organization, and the heterogeneity of neuronal responses to stimulus features beyond whisker identity, it is natural to ask whether selectivity to those features is also mapped, i.e. with neurons systematically distributed in space according to the stimulus parameters that they respond to. Recently, this question has been addressed at single-cell resolution by several calcium imaging studies that applied in vivo two-photon laser scanning microscopy to the upper layers of BC. These layers are a locus for integration for sensory inputs with those from other sources, and generate the main corticocortical output from BC.

### Organization of selectivity to spatial features

The first features to be explored with two-photon microscopy included spatial attributes of a tactile stimulus, such as whisker identity (Kerr et al., 2007, Sato et al., 2007, Clancy et al., 2015), the direction of whisker stimulation (Kerr et al., 2007, Kremer et al., 2011), and coherence of motion across whiskers (Estebanez et al., 2016).

At the macroscopic scale of barrel columns, whisker identity is mapped topographically. However, mapping is more variable at the single-neuron scale. The strength of the preference for the home whisker, i.e., the whisker corresponding to a neuron’s barrel column, can differ markedly for individual neurons within the same barrel column, even when located next to each other; moreover, some neurons may respond more strongly to a neighboring whisker, so that the actual principal whisker is not the one predicted by the neuron’s location (Sato et al., 2007, Clancy et al., 2015). Thus, diverse tuning as discussed above applies even to neurons located next to each other. This heterogeneity of response tuning from cell to cell recalls the so-called salt-and-pepper functional organization of visual orientation selectivity in the rodent primary visual cortex (Ohki et al., 2005, Mrsic-Flogel et al., 2007); rodent auditory cortex appears to share similar organization (Bandyopadhyay et al., 2010, Rothschild et al., 2010). Although this heterogeneity of responses applies throughout a barrel column, responses to whisker deflections tend to be more robust closer to the corresponding barrel center (Kerr et al., 2007).

Another spatial attribute of whisker stimulation is its direction or orientation. There has been interest in mapping directional selectivity for a long time. An electrophysiology study in anesthetized rats found “minicolumns” of neurons with identical directional selectivity within an individual barrel column (Bruno et al., 2003), but not a systematic relationship between the directional preference of a cell and its horizontal location within a barrel column. Later work based on electrophysiology and voltage-sensitive dye imaging did find a map of directional selectivity with respect to whisker barrel centers in BC, somewhat analogous to pinwheels in V1 (Andermann and Moore, 2006, Tsytsarev et al., 2010). In this finding, neurons located in the portion of a barrel column closest to another neighboring column tend to respond best to stimulation of their preferred whisker in the corresponding direction: for instance, neurons located in barrel C1 along the edge closest to C2 tend to respond preferentially to motion of whisker C1 toward C2 (Andermann and Moore, 2006). This map is not present in juvenile rats (Kerr et al., 2007) and emerges later in rat development (Wilson et al., 2010, Kremer et al., 2011). A map of directional selectivity has also been observed in awake mice performing active sensing (Peron et al., 2015), although with differences compared to that in the anesthetized rats: somatotopy seemed reversed in terms of selectivity to the lateral forces acting on a whisker. Moreover, neither that study (Peron et al., 2015) nor one in this Special Issue (Kwon et al., 2018) have found any topographical map for directional or orientation selectivity in anesthetized mice under passive stimulation. While this result is similar to findings in young anesthetized rats, it contrasts with older rats and awake, actively sensing mice. These differences could be partly attributable to species, but may also relate to differences in stimulation paradigm (use of cardinal axes (Peron et al., 2015, Kwon et al., 2018) vs. multiple directions (Andermann and Moore, 2006, Kerr et al., 2007, Kremer et al., 2011)). The mechanisms underlying directional selectivity maps merit further investigation.

How might directional selectivity be locally integrated across neurons and used? Directional tuning is progressively weakened along the pathway from PWAs to BC: while – as noted above – directional selectivity is present in BC, the information carried by single neurons about the direction of motion of their principal whisker decreases substantially from earlier stages to the cortical one (Bale and Petersen, 2009). However, the joint responses of pairs of BC neurons do convey on average just over twice the information about whisker direction carried by their constituent neurons (Bale and Petersen, 2009). Directional information could be obtained by jointly reading out responses from several BC neurons. Moreover, objects of a particular shape will induce patterns of changes in whisker motion direction across time and space: integration of directionally selective responses across neurons could be used to build a notion of object shape. Interestingly, in single neurons, tuning to global direction of “apparent motion” (sequential deflections across the whisker array) is independent from single whisker directional selectivity (Jacob et al., 2008). How populations combine these characteristics to construct codes for higher level properties such as shape remains an interesting and underexplored possible output for directional selectivity.

A third spatial feature of whisker stimuli is the degree of correlated motion across multiple whiskers. A recent study showed that layer 2/3 neurons in rat BC are systematically arranged in relation to this property of multiple-whisker motion (Estebanez et al., 2016). Neurons most responsive to uncorrelated stimulation tend to be located above barrels, while those preferring correlated or anti-correlated motion tend to be found over the edges of barrels. Thus, neurons selective to joint motion of the whisker predicted by the neuron’s location and a neighboring one tend to be found along the area partway between the corresponding barrel columns, presumably in a good position to receive convergent excitatory drive from both whiskers. The existence of similar maps in mice (where the septal area between barrel columns is not as much of a distinctive module as in rats) and their construction during development remain to be explored.

### Organization of selectivity to dynamical features

What about the spatial organization of selectivity to dynamical features in BC? Two-photon studies have determined the distribution of response selectivity for whisker angle and curvature in awake animals performing a pole localization task (Peron et al., 2015), kinematic features measured via passive stimulation in anesthetized animals (Martini et al., 2017), texture coarseness tested under electrical whisking (Garion et al., 2014), and distance to a wall contacted by the whiskers assessed in awake animals performing a virtual corridor-following task (Sofroniew et al., 2015). Although texture coarseness and distance to wall are not in themselves dynamical features, they can only be reconstructed from the dynamical properties of whisker motion during exploration of the texture or wall, and must reflect the evolution of more elementary features during contact.

Most of these studies, conducted in mice, found clear evidence favoring a salt-and-pepper distribution of feature selectivity, whether for tuning to kinematic features or wall distance (Peron et al., 2015, Sofroniew et al., 2015, Martini et al., 2017). Conversely, the study of selectivity to texture coarseness in rats found that neurons preferring the same texture tend to cluster together (Garion et al., 2014). Several differences between the studies may help explain this divergence. Differences in experimental condition include species, brain state (urethane anesthesia in the Garion study versus wakefulness or ketamine-xylazine anesthesia in the other studies), and stimulation paradigm (electrical whisking in the Garion study versus passive stimulation or active sensing in the other studies). There were also differences in how responses were measured, i.e., as a build-up during repeated stimulation (Garion et al., 2014) or a peak time-locked to a stimulus. Finally, important differences may relate to choices made during data analysis, leading to a positive detection of clusters in a topographical texture map (Garion et al., 2014) as compared to the more commonly found lack of clustering characteristic of the salt-and-pepper distribution (Peron et al., 2015, Sofroniew et al., 2015, Martini et al., 2017).

### Considerations on the spatial distribution of feature selectivity

A caveat to these studies is that they typically focus on a reduced set of parameters, covering a limited region of the rich stimulus space to which BC neurons are potentially responsive (Jacob et al., 2008, Estebanez et al., 2012, Maravall and Diamond, 2014). It is probable that each neuron participates in superimposed populations encoding different attributes of whisker-mediated sensory signals under different behavioral contexts, although essential properties such as touch (i.e., curvature-related signals) are likely to be present under most conditions. A further caveat is that analyses of spatial organization often focus on what parameter neurons are tuned to, rather than how strongly they are tuned. Even if a neuron passes a statistical test, quantitatively, the amount of information it conveys about some feature may be so low that it is functionally unimportant. A full picture of how populations are organized will need to consider the degree to which different neurons participate in conveying sensory and other relevant signals.

Pairs of neurons in mouse BC that share similar feature selectivity also have a higher noise correlation (stimulus-independent trial-to-trial correlations in their responses) (Kwon et al., 2018). This can arise as a result of the neurons sharing common inputs and/or being interconnected. Since such a set of neurons would be spatially interspersed with other sets with different feature selectivity, this suggests a scheme whereby neurons within a local region could be organized into interspersed subnetworks of preferentially connected and correlated cells. Such schemes have been found in the mouse visual (Ko et al., 2011, Cossell et al., 2015), auditory (Rothschild et al., 2010), and motor cortex (Komiyama et al., 2010) but need further exploration in BC.

More generally, the functional implications of the spatial layout of feature selectivity – mapped, clustered or salt-and-pepper – remain to be fully worked out. For example, how does the layout affect information transmission and decoding in downstream areas? One aspect of response heterogeneity in a local area, as found e.g., in a salt-and-pepper scheme, is that when information about a diversity of stimulus parameters is present within a local population of neurons, sampling across that population can enable robust downstream decoding of the sensory signal. This capacity for robust decoding is a property of population representations of texture in BC (Safaai et al., 2013).

## Organization of feature selectivity by cell type

First-order neurons in any sensory modality are endowed with sensitivity to particular stimulus features by virtue of the sensory receptors they express, their morphology and how they physically innervate the sensory organs. Neurons that differ in these properties will also differ in their feature selectivity. For example, in the whisker system, PWAs in the trigeminal ganglion innervate the vibrissa follicle sinus complex with terminations classified into several distinct morphological subtypes (reviewed in this Special Issue (Takatoh et al., 2018)). These confer sensitivity to different aspects of mechanical stimulation (Campagner et al., 2018). Despite the recent identification of the molecular identity of a subset of mechanoreceptors that play a role in whisker transduction, and of the specific feature selectivity of neurons expressing those receptors (Ikeda et al., 2014, Maksimovic et al., 2014, Woo et al., 2014, Severson et al., 2017), the correspondence between neuron and termination type and feature selectivity remains largely poorly known. This is a major challenge for understanding the nature of sensory-driven input to the whisker pathway.

The important notion that stimulus feature selectivity must be related to cell type also requires further exploration at higher stages of the whisker pathway. Differences in sensory responses were parsed by Simons and Carvell (1989) with respect to intrinsic electrophysiological response properties of excitatory and inhibitory neurons. Finer grained analyses of cell morphology, electrophysiological response properties, projection patterns and gene expression have conclusively demonstrated the existence of tens of distinct cell types at each stages of cortical processing, particularly GABAergic inhibitory neurons (Kepecs and Fishell, 2014, Harris and Shepherd, 2015, Poulin et al., 2016). While it is clear that inhibitory neurons are sensitive to different sensory and motor information than nearby excitatory neurons, even within excitatory neurons, distinct categories with different tuning properties are found when grouped across layers (De Kock et al., 2007, De Kock and Sakmann, 2009) or by projection target (Yamashita et al., 2013, Yamashita and Petersen, 2016, Chen et al., 2013, Chen et al., 2015, Harris and Shepherd, 2015). A general analysis of how cell type diversity maps onto feature selectivity remains to be conducted. Because the roles played by different cell types during behavior are likely to depend heavily on context – specific task performed, state of awareness and attention, etc. – this question may be best addressed during different types of whisker-mediated exploration in awake animals.

One potential use of diversity in feature selectivity is to segregate inputs into processing streams for a particular behavior (Glickfeld et al., 2013, Lur et al., 2016, Smith et al., 2017). A series of studies have investigated differences in the excitatory neurons that project from BC to M1 (M1P neurons) and to S2 (S2P neurons). These distinct populations of neurons – with strikingly little overlap – seem to convey different signals during awake behavior. M1P neurons appear to be important when whiskers contact a novel object – a behaviorally important situation that induces rapid changes in whisking strategy (Mitchinson et al., 2007, Grant et al., 2009, Deutsch et al., 2012). This reaction is possibly under the control of M1. M1P neurons have large spatial receptive fields as measured in anesthetized animals, i.e. they respond to a relatively broad set of whiskers (Sato and Svoboda, 2010, Clancy et al., 2015). Their responses correlate strongly with whisker stick–slip events (Chen et al., 2013) and they display fast and large synaptic potentials during passive deflections (Yamashita et al., 2013). In contrast, S2P neurons display different response properties than M1P neurons. In anesthetized animals, S2P neurons have whisker receptive fields with sharper resolution (Sato and Svoboda, 2010, Clancy et al., 2015). During active sensing, synaptic responses of M1P neurons to repetitive touch depress strongly, yet S2P neurons can fire consistently to subsequent touches (Yamashita et al., 2013). They can also display long-lasting depolarizations upon whisker stimulation during learned tasks (Yamashita and Petersen, 2016). Thus, S2P neurons, and their target pathway, may play an important role when whiskers make continuing surface interactions (Chen et al., 2013). Moreover, the activity of S2P neurons shows a stronger association with the animal’s choice in a perceptual detection task than other neurons in S1 (Kwon et al., 2016). While this activity is fed forward to S2, S2 feedback also transmits information about perceptual choice to S1, suggesting that S2P neurons may participate in intracortical feedback loops between these two areas (Kwon et al., 2016, Yang et al., 2016). Clearly, understanding the functional role of different processing streams during complex whisker-mediated tasks, and how this relates to coding properties of their constituent neurons, represents an important challenge for the field.

## Concluding remarks: outlook

Work on the structure and physiology of neuronal circuits across the whisker pathway has accumulated at an accelerating pace (Feldmeyer et al., 2013, Fox, 2018). How those neurons work together to generate whisker-mediated sensory behavior remains ill understood. Human introspection seems to afford little insight into the perceptual experience achieved by rodents using their whiskers; perhaps in part for this reason, the framework for exploring feature selectivity in neurons across the whisker pathway has lagged behind similar work in, for example, primate and carnivore vision.

Future work must explore how the sensitivity to elementary stimulus features found in neurons located subcortically and at early cortical stages is later integrated and combined, eventually generating percepts of object identity, shape, texture, distance, and motion. Current understanding of this key problem remains limited despite recent progress in capacities for tracking inputs to the system during natural behavior and for measuring the dynamics of neuronal activity linked to tactile events. Insights into how spatial and temporal patterns of whisker input are integrated by cortical neurons of defined types, and into how those patterns are reflected in ongoing cortical activity, will be crucial steps toward an explanation of whisker-mediated sensation. Such an explanation will likely require investigating the contributions of brain regions that are classically outside the somatosensory pathway, and accounting for differences between the forms of activity observed in different modes of whisker-based sensing. Two other related key directions for future work, as motivated above in this review, are the relation between the feature selectivity of a neuron and its circuit location and local connections; and the relation between the feature selectivity of a neuron and its cell type and long-range projection pattern. These microscopic principles of functional organization are likely to shape how information is streamed across the cortex.
